# Glutathione as a Molecular Marker of Functional Impairment in Patients with At-Risk Mental State: 7-Tesla ^1^H-MRS Study

**DOI:** 10.3390/brainsci11070941

**Published:** 2021-07-17

**Authors:** Peter Jeon, Roberto Limongi, Sabrina D. Ford, Cassandra Branco, Michael Mackinley, Maya Gupta, Laura Powe, Jean Théberge, Lena Palaniyappan

**Affiliations:** 1 Department of Medical Biophysics, Western University, London, ON N6A 3K7, Canada; yjeon4@uwo.ca (P.J.); jtheberge@lawsonimaging.ca (J.T.); 2 Lawson Health Research Institute, Imaging Division, London, ON N6A 4V2, Canada; 3 Robarts Research Institute, Western University, London, ON N6A 3K7, Canada; rlimongi@uwo.ca (R.L.); Sabrina.Ford@lhsc.on.ca (S.D.F.); Michael.MacKinley@lhsc.on.ca (M.M.); 4 Department of Psychiatry, Western University, London, ON N6A 3K7, Canada; cbranco3@uwo.ca (C.B.); Laura.Powe@lhsc.on.ca (L.P.); 5 Department of Neuroscience, Western University, London, ON N6A 3K7, Canada; 6 Department of Psychology, Western University, London, ON N6A 3K7, Canada; Maya.Gupta@lhsc.on.ca; 7 St. Joseph’s Health Care, Diagnostic Imaging, London, ON N6A 4V2, Canada; 8 Department of Medical Imaging, Western University, London, ON N6A 3K7, Canada

**Keywords:** psychotic disorders, schizophrenia, glutathione, magnetic resonance spectroscopy

## Abstract

A substantial number of individuals with clinical high-risk (CHR) mental state do not transition to psychosis. However, regardless of future diagnostic trajectories, many of these individuals develop poor social and occupational functional outcomes. The levels of glutathione, a crucial cortical antioxidant, may track variations in functional outcomes in early psychosis and prodromal states. Thirteen clinical high-risk and 30 healthy control volunteers were recruited for a 7-Tesla magnetic resonance spectroscopy scan with a voxel positioned within the dorsal anterior cingulate cortex (ACC). Clinical assessment scores were collected to determine if any association was observable with glutathione levels. The Bayesian Spearman’s test revealed a positive association between the Social and Occupational Functioning Assessment Scale (SOFAS) and the glutathione concentration in the clinical high-risk group but not in the healthy control group. After accounting for variations in the SOFAS scores, the CHR group had higher GSH levels than the healthy subjects. This study is the first to use 7-Tesla magnetic resonance spectroscopy to test whether ACC glutathione levels relate to social and occupational functioning in a clinically high-risk group and offers preliminary support for glutathione levels as a clinically actionable marker of prognosis in emerging adults presenting with risk features for various severe mental illnesses.

## 1. Introduction

Emerging adults with attenuated or brief and limited psychotic symptoms are said to be in a clinical high-risk (CHR) state (or “at-risk or ultrahigh-risk” mental state) that later develops into multiple diagnostic outcomes including schizophrenia, mood disorders, such as bipolar disorder, or a major depressive disorder [1,2]. A substantial number of individuals with CHR develop poor long-term functional (i.e., social and occupational) outcomes irrespective of diagnostic transitions. Longitudinal studies indicate that a large proportion of individuals with CHR do not transition to psychosis (65–89% not psychotic over 2–10 years [3,4,5]) but have poor social and occupational outcomes (48% functionally impaired at 3–10 years [3,6]). While functional outcomes improve over time in CHR patients who have good functioning at the baseline, persistent deficits are seen in those who start with lower levels of functioning [4]. In other words, lower levels of functioning in CHR state before the onset of diagnosable psychiatric disorders such as schizophrenia and bipolar disorders reliably predict the trajectory of continued poor functioning over a long time period. The molecular bases of such pervasive functional deficits continue to be unknown [7], proving to be a major hurdle in developing meaningful treatments aimed at CHR state.

Oxidative stress has emerged as a key mechanism underlying the pathophysiology of many psychiatric disorders including psychosis [8]. Destructive free radicals that damage brain tissue are by-products of oxidative metabolism but are effectively scavenged by antioxidants. Glutathione (GSH), the cardinal antioxidant in brain cells, shows 27–52% reduction [9,10,11] in established schizophrenia. Genetic [12,13] and cell biology studies [14,15,16] indicate that in a subset of patients, GSH production on demand is likely to be reduced [17]. We have recently demonstrated the prognostic importance of low GSH in predicting early clinical response to antipsychotics in first-episode schizophrenia. In this study, we observed that for every 10% baseline difference in anterior cingulate cortex (ACC) GSH among the patients, seven additional days of delay in response occurred after treatment initiation [18]. Lack of early response is a critical indicator of long-term poor outcomes in schizophrenia [19,20,21]. We and others have also related lower GSH to various determinants of functional outcomes including residual symptom burden [22], negative symptoms [23] and cognitive deficits [24], supporting the notion that the “hub of oxidative stress” indexed by GSH [25,26] is likely a critical determinant of functioning.

To date, various imaging modalities have been employed to predict both conversion to psychosis and functional outcomes in youth at CHR [27]. Structural MRI studies report pronounced cortical thinning [28,29] and longitudinal reduction in gray matter volume [30] in the ACC of CHR patients. These ACC abnormalities precede the psychosis onset [28,31]. Furthermore, aberrant activation of ACC in fMRI studies using tasks that demand executive controls has also been reported in CHR subjects who develop poor symptomatic and functional outcomes [32,33,34]. While several other distributed structural and functional changes precede and predict psychosis (see the review by Andreou and Borgwardt [35]), a recent meta-analysis has established that dorsal ACC (dACC) is the only brain region whose structural gray matter changes precede psychosis (i.e., in CHR state) and persist even after long periods of treatment (i.e., in both acute and chronic stages) [36], highlighting the crucial role of ACC for long-term outcomes.

We recently synthesized in vivo MRS studies on ACC and demonstrated a significant GSH reduction in the established cases of schizophrenia but an elevation in bipolar disorders [37], indicating that GSH levels may track the variations in functional outcomes that typify the prognostic course of psychiatric disorders. Such divergence between disorders may mean that in the “pluripotent” CHR state that includes patients with varying levels of functioning as a single group, GSH levels may not differ from healthy controls but relate to variations in levels of functioning. In fact, in the only previous MRS study of cortical glutathione in clinical high-risk state [38], da Silva et al. reported no difference between healthy controls and CHR subjects in anterior cingulate glutathione [39]. Functional outcomes were not evaluated in this study; thus, the role of GSH as a transdiagnostic prognostic marker in CHR state remains unknown.

In the current study, we use ultrahigh field 7T MRS for the first time to test if ACC GSH levels relate to social and occupational functioning in the CHR group. We expected GSH levels to be reduced among patients with poor functioning. Furthermore, we aimed at establishing the difference in GSH levels between the CHR and healthy control subjects after accounting for variations in functioning. We evaluated these hypotheses using Bayesian and frequentist statistical approaches.

## 2. Materials and Methods

### 2.1. Participants

We recruited 13 clinical high-risk (CHR) volunteers along with 30 healthy control (HC) volunteers group-matched for age, gender and parental socio-economic status. Patient volunteers were recruited from the referrals received by the PROSPECT (Prodromal Symptoms of Psychosis—Early Clinical Identification and Treatment) program at London Health Sciences Center, London, Ontario. The patients were help-seeking individuals referred to the clinic by community physicians, healthcare workers or friends/family. All the referrals were reviewed by an intake coordinator via telephone using a validated instrument (PRIME Screen—Revised). If found eligible for further assessment, the patients were evaluated within 2 weeks of referral using the Structured Interview for Psychosis-risk Syndromes (SIPS) [40]. Patients with medical conditions, pervasive developmental disorders or intellectual disability underlying the reported symptoms, those who received treatment with antipsychotic medications to treat presenting symptoms (minimal effective dose for a period of at least 2 weeks) and those with psychotic symptoms secondary to active substance use (intoxication effects) were excluded. Based on the SIPS, the patients satisfying attenuated psychotic syndrome (APS) or brief and limited intermittent psychosis (BLIPS) were both included in the CHR group. The healthy volunteers had no personal history of mental illness with no family history of psychotic disorder. All the participants were screened to exclude significant head injury, major medical illness or MRI contraindications and provided written informed consent according to the guidelines of the Human Research Ethics Board for Health Sciences at Western University, London, Ontario.

### 2.2. MRS Acquisition and Analysis

**Figure 1:**
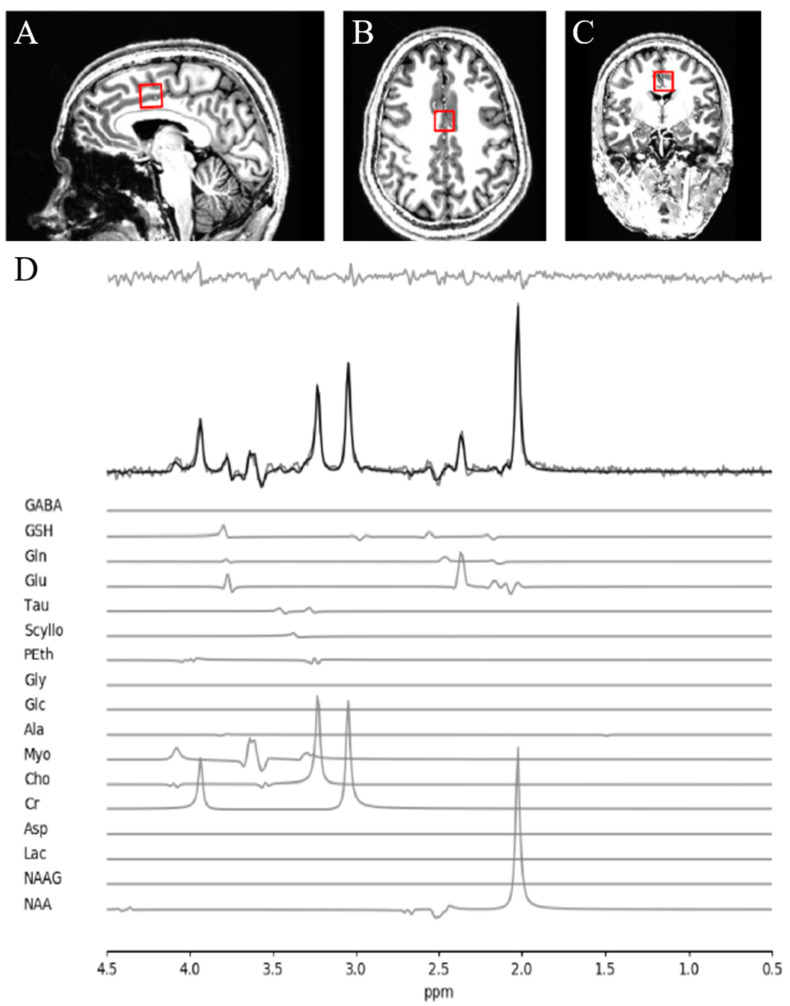
MRS voxel and spectra. (**A**) Sagittal, (**B**) axial and (**C**) coronal views of voxel positioning on the dorsal anterior cingulate cortex. (**D**) Sample spectra obtained from a single healthy participant. The bolded black line represents the fitted spectra with the residuals above and each individual metabolite contributions below.

A Siemens MAGNETOM 7T head-only MRI scanner (Siemens, Erlangen, Germany) was used for all MRS acquisition along with a site-built head coil (8-channel transmit, 32-channel receive coil array) at the Centre for Functional and Metabolic Mapping of Western University (London, ON, Canada). A two-dimensional sagittal anatomical image (37 slices, TR = 8000 ms, TE = 70 ms, flip-angle (*α*) = 120°, thickness = 3.5 mm, field of view = 240 × 191 mm^2^) was used as reference to prescribe a 2.0 × 2.0 × 2.0 cm^3^ (8 cm^3^) ^1^H-MRS voxel on the bilateral dorsal ACC (Figure 1). The voxel position was prescribed by setting the posterior face of the voxel to coincide with the precentral gyrus and setting the position of the inferior face of the voxel to the most caudal point not part of the corpus callosum. The voxel angle was set to be tangential to the corpus callosum. A semi-LASER ^1^H-MRS sequence (TR = 7500 ms, TE = 100 ms, bandwidth = 6000 Hz, *N* = 2048) was used to acquire 32 channel-combined, VAPOR [41] water-suppressed spectra as well as a water-unsuppressed spectrum to be used for spectral editing and quantification. All the participants were asked to fix their gaze on a white cross (50% gray background) during MRS acquisition.

Using the techniques outlined by Near et al. [42], the 32 spectra were phase- and frequency-corrected before being averaged into a single spectrum to be used for all subsequent analyses. QUECC [43] and HSVD [44] were applied to the spectrum for lineshape deconvolution and removal of the residual water signal, respectively. Spectral fitting was done using fitMAN [45], a time-domain fitting algorithm that uses a nonlinear iterative Levenberg–Marquardt minimization algorithm to echo time-specific prior knowledge templates. The metabolite fitting template included 17 brain metabolites: alanine, aspartate, choline, creatine, γ-aminobutyric acid (GABA), glucose, glutamate, glutamine, glutathione, glycine, lactate, myo-inositol, *N*-acetylaspartate, *N*-acetylaspartylglutamate, phosphorylethanolamine, scyllo-inositol and taurine. Due to the long echo time used, no significant macromolecular contribution was expected. Metabolite quantification was then performed using Barstool [46] with corrections made for tissue-specific (gray matter, white matter, CSF) T_1_ and T_2_ relaxation through partial volume segmentation calculations of voxels mapped onto T_1_-weighted images acquired using a 0.75-mm isotropic MP2RAGE sequence (TR = 6000 ms, TI_1_ = 800 ms, TI_2_ = 2700 ms, flip-angle 1 (*α*_1_) = 4°, flip-angle 2 (*α*_2_) = 5°, FOV = 350 mm × 263 mm × 350 mm, T_acq_ = 9 min 38 s, iPAT_PE_ = 3 and 6/8 partial k-space). All spectral fit underwent visual quality inspection as well as the Cramer–Rao lower bounds (CRLB) assessment for each metabolite.

The quality of metabolite quantification was measured using CRLB percentages for both groups using a CRLB threshold < 30% for glutathione to determine inclusion toward further analyses, in line with our prior study [18]. Notably, the mean CRLB for these metabolites were over two times lower than the individual threshold percentages. There was no significant difference in CRLB between the clinical high-risk group and the healthy controls for both metabolites reported in this study. We present the concentration and CRLB of other metabolites in our fitting template, along with the two presently mentioned, in the Table S1. A sample of fitted spectrum for a single participant is presented in Figure 1.

### 2.3. Clinical Assessments

Symptom severity was measured using the scale of prodromal symptoms (SOPS) on the same day of the scan. We also quantified the overall social and occupational functioning at the time of first presentation using the SOFAS [47] administered on the same day of the scanning. To determine cannabis use in the previous six months, the Cannabis Abuse Screening Test (CAST) was used [48]. The CAST is a six-item Likert-scale self-report questionnaire which asks the participant about cannabis use and how it affects their daily activities and relationships. Scores range from 6 to 30, with higher scores indicating more cannabis use. To determine alcohol use in the previous 6 months, the Alcohol Use Disorders Identification Test—Concise (AUDIT—C) [49] was used. The AUDIT—C is a three-item Likert-scale self-report questionnaire which asks the participant about alcohol use frequency and quantity. Scores range from 0 to 12, with higher scores indicating more alcohol use. Alcohol users and nonusers were classified by AUDIT—C scores of four or more and less than four, respectively. Lastly, nicotine use in the previous six months was determined using the single-item Fagerström Test for Nicotine Dependence and the smoking index [50]. The Fagerström test indicates time to the first cigarette after waking, and the smoking index is calculated by multiplying the number of years regularly smoking by the number of cigarettes per day divided by 20 cigarettes per pack. A lower Fagerström test value indicates more nicotine dependence, and a higher smoking index indicates more nicotine use. The 10-item Drug Abuse Screening Test (DAST-10) [51] was also employed for substances other than cannabis, alcohol and nicotine, though our cohort did not endorse any such use.

### 2.4. Bayesian Analysis

We evaluated the association between GSH and the SOFAS, the SOPS and the CAST scores in the CHR group and the relationship between GSH and the SOFAS scores in the HC group by using a Bayesian Spearman’s test [52]. This approach relies on data augmentation via the Metropolis-within-Gibbs sampling algorithm. Briefly, we assumed the rank data as the reflection of a latent (truncated) normal distribution which allowed us to use a conventional likelihood function. That is to say, the latent continuous scores would manifest as “degraded” rank values. Following this assumption, the data augmentation algorithm would yield samples from a truncated posterior distribution. Here, we tested the null hypothesis that ρ = 0 versus the alternative hypothesis that ρ ~ Uniform [−1,1] (i.e., following a uniform prior distribution). We drew 11,000 samples using a Markov chain Monte Carlo (MCMC) method using the “spearmanCorrelation.R” function in R as specified by van Doorn et al. [52]. We reported the Bayes factor relative to the null model (BF_10_). BF_10_ > 1.0 suggests evidence in support of the (alternative) hypothesis and vice versa. We also reported the mode and the proportion of the posterior distribution (i.e., posterior proportion, PP) of the estimated ρ (rho) values differing from zero along with the 95% highest density interval of the most credible values (HDI).

We estimated the posterior distribution of the (estimated) between-groups differences in the CAST, the AUDIT—C and the SOFAS scores by means of a generalized linear model (GLM) within the context of hierarchical Bayesian parameter estimation as follows:

$$scores_{i}=\beta_{0}+\underset{group}{\sum }\beta_{group}x_{group}\left(i\right)$$

where the data conformed to normal distribution around the predicted value (score) with (wide) data-scaled uniform prior distribution for the standard deviation (σ_i_). The baseline parameter ($\beta_{0})$ had a data-scaled normal prior distribution with the mean equal to the data mean and the (wide) standard deviation relative to the standard deviation (SD_data_) of the data (1/(SD_data_ × 5)^2^). Group deflection parameters ($\beta_{group})$ had normal prior distributions with mean zero and gamma prior distribution for the standard deviation σ_β_ with data-scaled shape and rate parameters (SD_data_/2 and 2 × SD_data_, respectively). This means that σ_β_ provided informed priors on each group (deflection) parameter. In other words, groups would act as priors between each other. In total, we estimated posterior distributions of five free parameters (σ_i_, $\beta_{0}$,$\;\beta_{HC}$,$\;\beta_{CHR}$ and σ_β_). The posteriors were estimated in RJAGS using MCMC, drawing 11,000 samples (thinning = 10). We reported the PP of the between-groups difference in scores.

To evaluate the group effect after accounting for the effect of the SOFAS scores, we included those scores as a covariate in the GLM,

$${\left[GSH\right]}_{i}=\beta_{0}+\underset{group}{\sum }\beta_{group}x_{group}\left(i\right)+\beta_{sofas}x_{sofas}\left(i\right)$$

in which we added a normal prior distribution of the covariate parameter $\left(\beta_{sofas}\right)$ which had zero mean and the data-scaled standard deviation equal to 1/(2 × SD_GSG_data/_SD_SOFAS_data_)^2^. In total, we estimated posterior distributions of six free parameters (σ_i_, $\beta_{0}$,$\;\beta_{HC}$,$\;\beta_{CHR}$, σ_β_ and $\beta_{sofas})$.

The posteriors were estimated in RJAGS using MCMC, drawing 11,000 samples (thinning = 10). We reported the PP of the between-groups difference in GSH along with the 95% HDI. The posterior distribution of the effect size of this difference is also reported. There were two reasons for employing a Bayesian approach. First, we were interested in recovering the whole posterior distribution of possible values of the parameters given the data, constraining our discussion to the 95% highest density interval. Second, a Bayesian approach allowed us to identify the support not only for the alternative hypothesis, but also for the null hypothesis.

### 2.5. Frequentist Analysis

All frequentist statistical tests were computed using IBM SPSS Statistics version 26 [53]. Group demographic differences were calculated using *t*-tests and chi-squared tests for continuous and dichotomous variables, respectively. Hierarchical regression was used to assess the effect of the SOFAS scores and diagnosis (dummy coded CHR = 0, healthy controls = 1), with parameter estimates examined to test individual variable effects. Lastly, Spearman’s correlation was used to determine the correlation between metabolite levels and clinical scores (SOFAS, CAST, AUDIT—C, SOPS).

## 3. Results

### 3.1. Demographic Data

Subject demographic and clinical data are summarized in Table 1. A small number within the clinical high-risk group were being administered antidepressants (*N* = 3) or benzodiazepine (*N* = 2) at the time of the scan. Mean percent CRLB values for CHR and HC GSH were 10 ± 4% and 11 ± 4%, respectively. The CHR patients had substantially high levels of functional impairment. The groups differed in the CAST scores, which were higher in the CHR group than in the HC group (mode = 4.19, posterior proportion (PP) = 1.0), but not in the AUDIT—C scores (mode = −0.01, PP = 0.6). The SOFAS scores were higher in the HC group than in the CHR group (mode = 16.7, PP = 1.0).

### 3.2. GSH, CHR Status and Social and Occupational Functioning

The Spearman’s test revealed a positive association between the Social and Occupational Functioning Assessment Scale (SOFAS) scores and GSH in the CHR group (mode ρ = 0.58, posterior proportion (PP) = 0.98, Bayesian factor in favor of H_1_ over null H_0_ (BF_10_) = 2.1), whereas in the HC, the test indicated “absence of association” (mode ρ = 0.11, PP = 0.44, BF_10_ = 0.23). In the CHR group, there was neither an effect of the SOPS (mode ρ = −0.17, PP = 0.78, BF_10_ = 0.22) nor an effect of the CAST (mode ρ = 0.32, PP = 0.87, BF_10_ = 0.46) on GSH (Table 2). Note that most healthy controls reported no cannabis use, leading to limited variance in the CAST scores that could not be meaningfully related to GSH.

**Figure 2:**
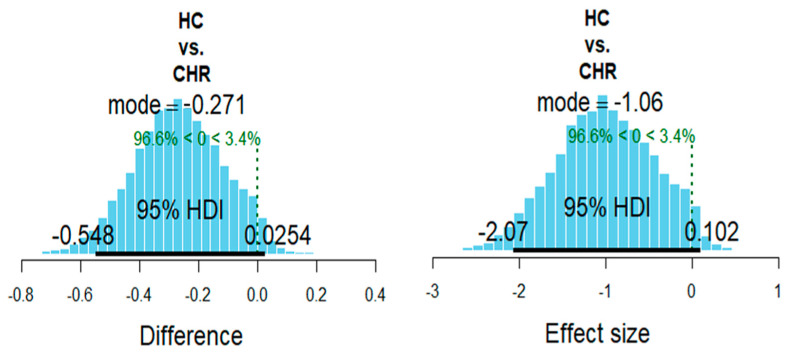
Bayesian analysis. Posterior distributions of the estimated between-groups difference in glutathione.

After accounting for the SOFAS scores, the metabolite level in the HC group was smaller than in the CHR group (mode difference = −0.27, PP = 0.97; effect size −1.06, PP = 0.97). Summary statistics of the posterior distributions of the model’s parameter estimates are reported in Table 2. Figure 2 shows the posterior distributions of the estimated between-groups difference in GSH.

### 3.3. Frequentist Analysis

**Figure 3:**
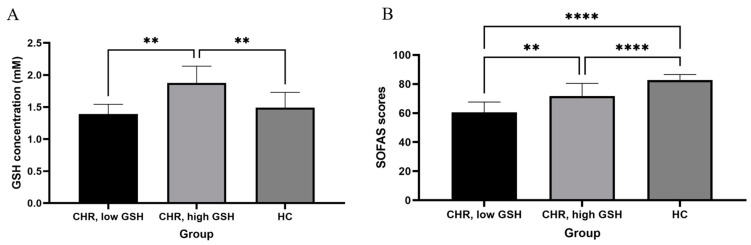
Median split analysis of glutathione (GSH) on the SOFAS. (**A**) Mean (± SD) glutathione concentrations (mM) of the low-GSH (< 1.60 mM; *N* = 7) and high-GSH (> 1.60 mM; *N* = 6) subgroups of clinical high-risk (CHR) cohorts as well as of the healthy controls (HC; *N* = 30). (**B**) Mean (± SD) SOFAS scores of the same three groups. Asterisks denote significant differences between the groups based on one-way ANOVA with the Tukey’s multiple comparison test, with ** denoting *p* < 0.05 and **** denoting *p* < 0.001.

With the positive association between the SOFAS scores and GSH already established in CHR using Bayesian analysis, our frequentist analysis was performed as a replication rather than as an addition. Median splitting of glutathione concentrations in the patient group (Figure 3) revealed a significant difference between the SOFAS scores of the low-glutathione (< 1.60 mM) and high-glutathione (> 1.60 mM) subgroups (t(11) = −2.49, *p* = 0.03), consistent with the Bayesian results relating GSH to the SOFAS scores in the CHR group.

In a hierarchical regression model with the GSH level as the dependent variable, with the SOFAS score entered as a predictor for all the subjects, the adjusted R^2^ of the model was −0.024, F(1, 41) = 0.013, *p* = 0.9, an insignificant effect. When the CHR status was included in the model, the R^2^ of the model increased to 0.07. This R^2^ increase was statistically significant, F(1, 40) = 5.18, *p* = 0.028. The regression coefficient for the CHR status was significantly negative (B = −0.56, t = −2.28), indicating that the GSH level was significantly higher in CHR subjects than in healthy controls after controlling for variance due to SOFAS. In this model, the SOFAS had a trend level of significance as a predictor, indicating that higher SOFAS scores are seen in the presence of higher GSH levels (B = 0.43, t = 1.75, *p* = 0.09). These results are in keeping with the Bayesian analysis reported in the manuscript.

## 4. Discussion

Our data provide evidence in support of a relationship between GSH levels and social and occupational functioning in clinical high-risk state and the presence of higher GSH in CHR subjects when the variance related to functional impairment is accounted for. In this study, we observed no relationship between GSH levels in the ACC and the SOFAS in the healthy volunteers, especially as the functional variability was within a narrow range among the healthy subjects. Furthermore, we did not observe any correlations between GSH and prodromal positive symptom severity. Taken together, these results support our hypothesis that GSH is a key molecular substrate underlying the functional deficits seen in the CHR state.

We report higher GSH after accounting for differences in functioning in CHR compared to healthy subjects, with reduced GSH being associated with reduced functioning among the CHR subjects. In the presence of CHR, higher levels of GSH may occur as a compensatory process across the spectrum (thus leading to a group level increase in GSH), but those who do not mount a sufficient GSH response may have poorer functional outcomes. In healthy subjects, this compensatory drive does not occur. In line with our prior work demonstrating the potentially counteracting effect of GSH on glutamatergic excitotoxicity in early stages of psychosis [54,55], we only examined the dACC in this study. GSH levels in the dACC also relate to core symptoms of psychosis such as disorganization [56], which strongly predict poor outcomes when present in CHR [57]. Nevertheless, antioxidant aberrations related to psychosis are unlikely to be limited only to the dACC, as striatal and thalamic reductions of GSH were also demonstrated in prior MRS studies [24,58].

An exciting translational utility of identifying GSH deficit in low-functioning patients is the therapeutic possibility of correcting it. A number of compounds with the potential to correct the effects of GSH deficit are in the pipeline [59,60,61]. Of these, *N*-acetylcysteine has been shown to improve cognition and negative symptoms in schizophrenia (six RCTs) [62] and global functioning in mood disorders [63,64]. Given the persistent nature of functional deficits, reversing them will likely require longer trials that are substantially difficult to complete. Antioxidants that increase GSH levels are more likely to benefit patients whose GSH levels are lower to begin with [65]. Our results support stratifying antioxidant trials on the basis of the baseline functional impairment or GSH levels in the future.

Our study has a number of strengths. We used the 7T-MRS sequence with improved specificity to detect GSH resonance with reduced macromolecular interference [18,66]. Among the MRS studies specifically optimized for GSH detection, 7T studies [22,67] report higher effect sizes for GSH reduction in schizophrenia compared to 3T [12,68]. We also recruited patients who had not been treated with antipsychotics and evaluated an age-, gender- and parental socio-economic status-matched control group. Nevertheless, our sample size was limited compared to the prior study addressing this issue using a 3T-MRS sequence. Furthermore, we lacked the follow-up data necessary to identify transition to psychosis among the CHR groups. From the published meta-analytical data, we expect from one to three converters in the next 2–5 years of observation [69]. The final limitation of our work was the imbalance of certain demographic characteristics, namely, CAST score differences between groups and the imbalance of gender within the CHR group.

## 5. Conclusions

In summary, our data offer preliminary support for the GSH level as a clinically actionable marker of prognosis in emerging adults presenting with risk features for various severe mental illnesses. The use of a longitudinal approach to track GSH levels in future CHR studies may help establish the mechanistic primacy of the antioxidant status in determining long-term outcomes.

## Figures and Tables

**Table 1:** Demographics and clinical characteristics.

Characteristics	Clinical High Risk (*N* = 13)	Healthy Controls (*N* = 30)	*t/χ^2^*	*p*
Gender (male/female)	11/2	19/11	1.948	0.163
Marital status (Mar/S)	1/12	2/28	0.009	0.926
Age (M/SD)	22.2/4.0	21.8/3.7	−0.330	0.745
Parental NS-SEC (M/SD)	3.54/0.88	3.03/1.38	−1.444	0.158
Cannabis use (Y/N)	6/7	1/29	4.926	0.026
Antidepressant use (Y/N)	3/10	-		
Benzodiazepine use (Y/N)	2/11	-		
Antipsychotic use (Y/N)	1/12	-		
SOPS, total (M/SD)	8.4/5.0	-		
CAST, total (M/SD)	11.4/7.8	6.2/0.8	−2.20	0.052
Audit—C, total (M/SD)	5.5/3.8	5.9/2.5	0.281	0.783
SOFAS (M/SD) APS/BLIPS Converted (Y/N)	67.7/9.5 13/0 3/10	82.7/3.8	6.267	0.000

Chi-squared analyses for categorical variables and independent *t*-tests for continuous variables were used to calculate *p*-values for differences between groups. Mar—married, S—single, M—mean, SD—standard deviation, NS-SEC—National Statistics Socio-economic Classification, Y—yes, N—no, SOPS—scale of prodromal symptoms, CAST—Cannabis Abuse Screening Test, AUDIT—C—Alcohol Use Disorders Identification Test—Concise, SOFAS—Social and Occupational Functioning Assessment Scale, APS—attenuated psychotic syndrome, BLIPS—brief and limited intermittent psychosis.

**Table 2:** Parameter estimates (posteriors) of the hierarchical Bayesian linear model and the Spearman’s correlation test.

Parameter	Mean	Median	Mode	HDI_low_	HDI_high_
$\beta_{0}$	0.849	0.842	0.823	−0.165	1.875
*β*_[CHR]_	0.135	0.135	0.135	−0.013	0.274
*β*_[HC]_	−0.135	−0.135	−0.135	−0.274	0.013
*β*_SOFAS_	0.009	0.010	0.010	−0.004	0.023
σ_β_	0.440	0.335	0.199	0.000	1.155
σ_i_	0.270	0.267	0.264	0.212	0.332
ρ_[HC]SOFAS_	0.154	0.143	0.114	−0126	0.378
ρ_[CHR]SOFAS_	0.478	0.509	0.586	0.045	0.769
ρ_[CHR]SOPS_	−0.073	−0.076	−0.171	−0.335	0.196
ρ_[CHR]CAST_	0.201	0.207	0.315	−0.124	0.500

Note: HDI represents 95% of the most credible values. HDI—highest density interval, $\beta_{0}$—intercept, *β*_[CHR]_—deflection parameter for the CHR group, *β*_[HC]_—deflection parameter for the HC group, *β*_SOFAS_—deflection parameter for the SOFAS covariate, σ_β_—standard deviation of the baseline parameter, σi—standard deviation of the predicted value, ρ_[HC]SOFAS_—Spearman’s correlation between the SOFAS score and GSH of the control group, ρ_[CHR]SOFAS_—Spearman’s correlation between the SOFAS score and GSH of the CHR group, ρ_[CHR]SOPS_—Spearman’s correlation between the SOPS score and GSH of the CHR group, ρ_[CHR]CAST_—Spearman’s correlation between the CAST score and GSH of the CHR group.

## Data Availability

The data presented in this study are available on request from the corresponding author.

## Supplementary Materials

The following are available online at https://www.mdpi.com/article/10.3390/brainsci11070941/s1, Table S1: Mean metabolite concentration (SD) and mean CRLB (SD).
