# Supplementary material for: Glutathione as a Molecular Marker of Functional Impairment in Patients with At-Risk Mental State: 7-Tesla 1H-MRS Study
Source: Brain Sci. 2021 Jul 17;11(7):941. doi: 10.3390/brainsci11070941 (PMC8307096; doi:10.3390/brainsci11070941)
Supplement: Supplementary file 1 [file brainsci-11-00941-s001.zip › brainsci-1193724-supplementary.pdf]

Supplementary Materials

Metabolite Profiles

Out of 17 neurometabolites included in the spectral fitting template, eight metabolites passed the ≤ 45% individual CRLB threshold (Table S1).

Table S1. Mean metabolite concentration (SD) and mean CRLB (SD).

	**[CHR]**	**$CRLB_{CHR}$**	**[HC]**	**$CRLB_{HC}$**
**NAA**	10.81 (0.96)	1.01 (0.23)	10.36 (1.16)	1.01 (0.41)
**Choline**	2.51 (0.32)	1.99 (0.42)	2.45 (0.27)	1.85 (0.53)
**Creatine**	8.96 (0.84)	1.35 (0.22)	8.48 (0.88)	1.29 (0.30)
**Glutamate**	6.66 (0.77)	3.74 (0.74)	6.56 (0.88)	3.38 (0.97)
**Glutamine**	1.02 (0.27)	22.84 (8.26)	1.05 (0.28)	19.62 (6.59)
**Glutathione**	1.62 (0.32)	10.95 (3.74)	1.49 (0.24)	10.56 (3.63)
**Myo-inositol**	4.76 (0.68)	4.47 (0.85)	4.72 (0.69)	4.08 (1.13)
**Scyllo-inositol**	0.31 (0.14)	24.53 (11.49)	0.30 (0.12)	20.27 (6.74)
**Taurine**	1.16 (0.44)	27.24 (8.34)	1.10 (0.43)	25.27 (7.80)

SD standard deviation, *CRLB* Cramer-Rao lower bound, *CHR* clinical high-risk, *HC* healthy controls, *NAA* N-acetyl aspartate

Note: Mean (SD) concentration and CRLB units are measured in mM and %, respectively. Only those metabolites with CRLB ≤ 45% were included in this table (all CRLB outliers of ≥ 45% were removed). As a result, HC glutamine, scyllo-inositol, and taurine had N = 29 while CHR glutamine, myo-inositol, and scyllo-inositol had N = 12, and CHR taurine had N = 11. All other HC (N = 30) and CHR (N = 13) metabolites listed included the whole participant pool. All other metabolites in our fitting template not listed in this table were due to poor CRLB measurements.

Supplementary Table Captions

Table S1. Mean metabolite concentration (SD) and mean CRLB (SD).
